# Childhood Nephrotic Syndrome Complicated by Catastrophic Multiple Arterial Thrombosis Requiring Bilateral Above-Knee Amputation

**DOI:** 10.3389/fped.2020.00107

**Published:** 2020-03-19

**Authors:** Hayato Togashi, Yuko Shimosato, Ken Saida, Noriko Miyake, Takeshi Nakamura, Shuichi Ito

**Affiliations:** ^1^Department of Pediatrics, Saiseikai Yokohamashi Nanbu Hospital, Yokohama, Japan; ^2^Department of Pediatrics, Yokohama City University Graduate School of Medicine, Yokohama, Japan; ^3^Department of Human Genetics, Yokohama City University Graduate School of Medicine, Yokohama, Japan; ^4^Department of Rehabilitation Medicine, Yokohama City University Graduate School of Medicine, Yokohama, Japan

**Keywords:** nephrotic syndrome, arterial thrombosis, *PROS1* gene, protein S, amputation

## Abstract

**Background:** Thromboembolic events are rare but critical complications in childhood nephrotic syndrome. The veins are more commonly affected, while arterial thrombosis is extremely rare but often life-threatening. Herein, we describe the clinical course of a 10-years-old girl with catastrophic multiple arterial thrombosis at the primary onset of nephrotic syndrome who underwent bilateral above-knee amputation.

**Case diagnosis/treatment:** A previous healthy 10-years-old girl contracted the influenza B virus. Five days later, she suddenly developed severe ischemia in both legs. Physical examination showed eyelid and leg edema, and laboratory tests revealed hypoalbuminemia and acute kidney injury. After undergoing contrast-enhanced computed tomography, the patient was diagnosed with multiple arterial thrombosis (including the bilateral iliac arteries) due to nephrotic syndrome. Despite the performance of surgical thrombectomies, fasciotomy, and systematic heparinization, she required bilateral above-knee amputation. The patient achieved spontaneous remission of nephrotic syndrome, and her renal function fully recovered. There were no findings suggestive of secondary nephrotic syndrome and antiphospholipid syndrome. Her protein C and protein S concentrations were slightly decreased at admission. However, whole-exome sequencing revealed a thrombotic risk variant (T630I) in the *PROS1* gene encoding protein S. This missense variant is often reported in patients with thrombosis or protein S deficiency, and may result in a thrombotic predisposition in some situations, such as nephrotic syndrome.

**Conclusions:** Arterial thrombosis is a rare complication; however, it must be considered, especially in patients with new-onset nephrotic syndrome. Early recognition is important for early intervention and prevention of serious sequelae.

## Introduction

Thromboembolism is a well-known complication of nephrotic syndrome (NS). The suggested risk factors for thromboembolic complications in children with NS include urinary loss of anticoagulants, increased synthesis of clotting factors, alterations of the fibrinolytic system, thrombocytosis, enhanced platelet activation and aggregation, and blood hyperviscosity due to hemoconcentration (1–3). The incidence of thromboembolic complications in children with NS is reportedly 1.8–4.4% (4). Veins are most commonly affected (1, 4). In contrast, arterial thrombosis is extremely rare, but often becomes serious (4). Herein, we describe a case of primary-onset childhood NS complicated by catastrophic multiple arterial thrombosis that required bilateral above-knee amputation.

## Case Presentation

A previously healthy 10-years-old girl was transferred to our intensive care unit because of severe ischemia in both legs. She had contracted the influenza B virus 1 week before admission. After 5 days of abdominal pain and vomiting, she had suddenly developed severe pain and cyanosis in both legs on the morning of the day of admission. She was diagnosed with multiple thrombosis based on computed tomography findings at the previous hospital. Physical examination at the time of admission showed eyelid and leg edema and severe cyanosis of both legs. Both legs below the inguinal region had become dark purple-colored and were cold to touch. The bilateral popliteal and dorsalis pedis pulses were markedly diminished. The patient was unable to move due to severe leg pain. The onset of severe pain and cyanosis in both legs had occurred more than 10 h prior to admission.

On admission, she had a blood pressure of 133/88 mmHg, pulse of 100 beats per minute, and respiratory rate of 22 breaths per minute. Laboratory test results were: urine protein-to-creatinine ratio, 10.7 g/gCr (reference: <0.15g/gCr); serum albumin, 1.0g/dL (reference: 4.1–5.1g/dL); total cholesterol, 465mg/dL (reference: 142–248 mg/dL); urea nitrogen, 68mg/dL (reference: 8–20mg/dL); serum creatinine, 0.89mg/dL (reference: 0.30–0.57mg/dL); uric acid, 12.1mg/dL (reference: 2.6–5.5 mg/dL); creatinine kinase, 3,858IU/L (reference: 41–153IU/L); sodium, 117 mEq/L (reference: 138–145mEq/L); potassium, 6.4 mEq/L (reference: 3.6–4.8mEq/L); fibrinogen degradation products, 447 μg/mL (reference: <5 μg/mL); D-dimers, 11.48 μg/mL (reference: <0.70 μg/mL); antithrombin III, 66% (reference: 78–125%). Laboratory abnormalities suggested NS, acute kidney injury, and rhabdomyolysis.

**Figure 1:**
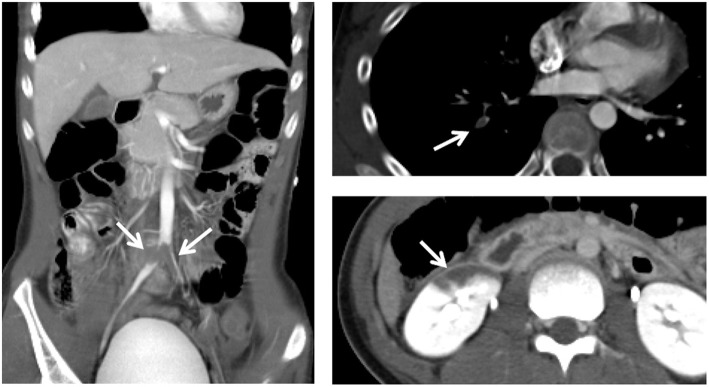
Contrast-enhanced computed tomography reveals multiple thrombi (arrows) in the bilateral common iliac, external iliac, and femoral arteries, right pulmonary artery, and right renal artery.

Contrast-enhanced computed tomography revealed multiple thrombi in the bilateral common iliac, external iliac, and femoral arteries, right pulmonary artery, and right renal artery (Figure 1).

Since cyanosis had developed more than 10 h before presentation, there was concern that thrombolytic therapy would not be effective due to the likelihood of formation of organized thrombi. Therefore, urgent surgical thrombectomy was performed from the external iliac arteries. Partial removal of thrombi was achieved, but the distal thrombi could not be removed. Most of the thrombi were organized and there were few fresh thrombi, which suggested that the best time to perform thrombolytic therapy had already passed. The patient subsequently developed compartment syndrome in both legs, which required urgent fasciotomy on the day of admission. During surgical thrombectomy, hemodiafiltration was started in the operation room because of anuria due to acute kidney injury and progressive rhabdomyolysis.

Systematic heparinization was initiated. Prednisolone was not started because of the increased risks of thrombosis, infection, and the difficulty in evaluating proteinuria in an anuric patient. Although the popliteal pulses slightly recovered in both legs after surgical thrombectomy, the patient required a second thrombectomy using a Fogarty catheter on the 4th hospital day due to weakened bilateral popliteal and dorsalis pedis pulses. Bilateral above-knee amputations were performed on the 6th hospital day because of the progression of necrosis and suspected infection. Urination had begun and hemodialysis was withdrawn on the 23rd hospital day. The urine protein-to-creatinine ratio spontaneously decreased, and the patient achieved complete remission of NS on the 44th hospital day. Her renal function also fully recovered. Despite the heparinization, ultrasonography showed a thrombus in the abdominal aorta, which necessitated the addition of clopidogrel on the 26th hospital day. However, an iatrogenic pseudoaneurysm in the right brachial artery was found, and so antithrombotic and antiplatelet agents were discontinued on the 31st hospital day. Contrast-enhanced computed tomography revealed a thrombus in the right internal jugular vein on the 66th hospital day, which required warfarin therapy. The patient was discharged on day 81. All thrombi had disappeared, and warfarin was withdrawn at 2 months after discharge. The patient is currently able to walk using bilateral artificial legs, and has shown no signs of recurrence of NS and/or thrombosis.

Further evaluation was undertaken to determine the underlying etiology of the arterial thrombosis. Laboratory testing performed while the patient was in the state of NS showed a low antithrombin III level of 66% (reference: 78–125%), low protein C activity of 67% (reference: 70–150%), and low protein S level of 56.0% (reference: 63.5–149.0%). Factor VII level was normal. Complement study revealed that the C3, C4, and CH50 levels were within normal limits. Tests for antinuclear antibody, anti-double stranded DNA antibody, lupus anticoagulant, anticardiolipin antibody, and anti-neutrophilic cytoplasmic antibody were all negative. Whole-exome sequencing revealed negativity for factor V Leiden and prothrombin G20210A, which are well-known causes of thrombosis, but showed a missense variant in the *PROS1* gene encoding protein S (NM_000313.3: c.1889C>T: Thr630Ile), which is recurrently reported in patients with thrombosis. Thus, we are considering restarting anticoagulant therapy.

## Discussion

The frequency of thromboembolic complications in children with NS is reported to be between 1.8 and 4.4% (4). The hypercoagulable state is multifactorial, attributed predominantly to urinary loss of anticoagulants, increased procoagulatory activity, impaired fibrinolysis, thrombocytosis, platelet hyperaggregability, and blood hyperviscosity due to hemoconcentration (1–3). Thrombotic complications are predominantly venous, but arterial thrombosis can also occur and often becomes serious. A previous single-center study reported 35 thromboembolic events in 34 children with NS over a period of 7 years, including cerebral venous thrombosis (*n* = 11), pulmonary thromboembolism (*n* = 9), deep vein and superior vena cava thrombosis (*n* = 6), intracranial arterial thrombosis (*n* = 7), and peripheral arterial thrombosis (*n* = 2) (4). The common sites of arterial thrombosis are the cerebral, pulmonary, and femoral arteries, where turbulent flow occurs from arterial branching (4, 5). Arterial thrombosis has also been reported at other sites, such as the abdominal aorta and the renal, mesenteric, and brachial arteries (6).

According to previous reports, thromboembolic events often develop within months of diagnosis and during treatment of NS (7). The risk factors for thromboembolic complications in children with NS are: an age of over 12 years, thromboembolic history preceding the diagnosis of NS, secondary NS, and membranous nephropathy (7). The risk of thromboembolic complications is also increased by dehydration, infection, immobilization, arterial or venous puncture, history of diuretic, or corticosteroid use, presence of hemoconcentration (hemoglobin >14 g/dL), thrombocytosis (>450 × 10^9^ platelets/L), severe proteinuria, hypoalbuminemia (<2.0 g/dL), hyperfibrinogenemia, and hypoantithrombinemia (<75%) (1, 3, 4, 8–10). In our case, the bedrest and decreased water intake during influenza B virus infection could have accelerated the thrombogenesis; other risk factors for thrombosis included hypoalbuminemia, hyperfibrinogenemia, and decreased levels of antithrombin III, protein C, and protein S.

Table 1 summarizes 11 cases of arterial thrombosis in the extremities in pediatric idiopathic NS, including the present case (8–17). The median age at the time of the thrombotic event was 8 years (range 1–15 years). Arterial thrombosis occurred during the first episode of NS in five of eight cases (62.5%), and corticosteroids were used in seven of 11 cases (63.6%). Hypoalbuminemia (<2.0 g/dL) was observed in five of eight cases, including our case. Surgical thrombectomies were performed in nine of 10 cases (90.0%), and amputations were performed in six of 11 cases (55.6%).

**Table 1:** Published cases of arterial thrombosis of the extremities in pediatric idiopathic nephrotic syndrome.

**References**	**Cameron et al. (11)**	**Harrison and Wood (12)**	**Parrish et al. (8)**	**Maffei et al. (13)**	**Tarry et al. (9)**	**Farkas et al. (10)**	**Büyükçelik et al. (14)**	**Koh et al. (15)**	**Han et al. (16)**	**Chinnadurai et al. (17)**	**Our case**
Age/sex	1/F	3/M	8/M	1/M	15/M	15/M	14/F	15/N/A	2/F	8/M	10/F
Thromboembolic site	Popliteal artery	Femoral artery	Common iliac, external iliac, and femoral arteries	Femoral artery	Brachial, ulnar, and radial arteries	Popliteal artery	Femoral and posterior tibial arteries	Femoral and popliteal arteries	Common iliac, external iliac, and popliteral arteries	Posterior tibial and peroneal arteries	Common iliac and femoral arteries
Episode	N/A	N/A	Relapse	First	First	Relapse	First	Relapse	First	First	First
Steroid administration	Yes	Yes	Yes	No	No	Yes	No	Yes	Yes	Yes	No
Steroid sensitivity	N/A	Resistant	Sensitive	Sensitive	N/A	Resistant	Resistant	Sensitive	Resistant	Sensitive	–
Type of nephrotic syndrome	N/A	MPGN	MC	MC	N/A	MC	MN	MC	FSGS	FSGS	–
Albumin (g/dL)	N/A	N/A	0.8	N/A	0.4	1.8	2.2	3.8	2.5	1.4	1.0
Fibrinogen (μg/dL)	N/A	N/A	N/A	N/A	N/A	630	381	N/A	362	N/A	949
Antithrombin III (%)	N/A	N/A	N/A	N/A	N/A	65	83	N/A	91	46	66
Thrombectomy	Yes	N/A	Yes	Yes	Yes	Yes	No	Yes	Yes	Yes	Yes
Thrombolysis	Yes	N/A	No	No	Yes	No	No	No	No	Yes	No
Fasciotomy	No	N/A	No	No	Yes	No	No	Yes	No	Yes	Yes
Amputation	Femur	N/A	Femur	No	Digits of the hand	Lower leg	No	No	No	Digits of the foot	Bilateral femurs

*N/A, not available; MPGN, membranoproliferative glomerulonephritis; MC, minimal change; MN, membranous nephropathy; FSGS, focal segmental glomerulosclerosis*.

The therapeutic approach to arterial thrombosis in children includes surgical intervention, systematic anticoagulation, and thrombolytic therapy. Tissue plasminogen activator is reportedly effective in pediatric patients; however, its risk-benefit ratio remains unclear (4). Our patient could not undergo thrombolysis therapy because it had been more than 10 h since the onset of leg pain and cyanosis. Therefore, surgical thrombectomy was performed. As the removed thrombi were almost all organized, thrombolysis therapy was unlikely to have been effective. In our patient, it is likely that organized chronic thrombi had disseminated from the bifurcation site of the common iliac artery and had occluded the femoral artery. Despite multiple interventions including surgical thrombectomies, fasciotomy, and systematic heparinization, our patient regretfully required bilateral above-knee amputation to save her life.

Our patient achieved spontaneous remission of NS and showed no recurrence of NS. The cause of NS in the present case remains unclear without a kidney biopsy, but was presumably due to minimal change NS. Spontaneous remission of minimal change NS has been reported in cases complicated by viral infections such as measles, varicella, and influenza B (18–20). Additionally, trauma induces inflammation, but counter-immunosuppression subsequently develops. There is an increased risk of counter-immunosuppression in injured patients. Such immunosuppression after trauma can induce spontaneous remission of NS (21, 22). The severe clinical course in our patient, including both viral infections and trauma, may have led to the spontaneous remission.

Investigations into predisposition for thrombosis should be performed in patients with severe or multiple thrombosis. Our patient had no past medical history and/or family history of thromboembolic complications. There were also no findings suggestive of secondary NS, protein C and protein S deficiency, and antiphospholipid syndrome. Whole-exome sequencing revealed a risk variant in the *PROS1* gene. This missense variant is rare in the general population, but is often reported in patients with thrombosis or protein S deficiency (23, 24). Protein S acts as a cofactor to activated protein C (APC) in the degradation of factor Va and factor VIIIa (25, 26). Approximately 60% of the protein S in human plasma is conjugated with C4b-binding protein, resulting in the complete loss of APC cofactor activity (26). *PROS1* variants can affect the secretion of protein S, APC cofactor activity, and inhabitation by C4b-binding protein. A prior study revealed protein S activity and antigen were not markedly decreased in this mutation (24). Although its pathogenicity is not clear, this missense variant could have been one of the risk factors for thrombosis in our patient.

Arterial thrombosis is a rare complication of NS that must be considered in patients with new-onset NS. Early recognition is important in order to initiate treatment promptly and prevent serious sequelae.

## Data Availability Statement

The raw data supporting the conclusions of this article will be made available by the authors, without undue reservation, to any qualified researcher.

## Ethics Statement

Written informed consent was obtained from the minor(s)' legal guardian/next of kin for the publication of any potentially identifiable images or data included in this article.

## Author Contributions

HT, YS, TN, and SI assumed clinical duties for this patient. HT and SI reviewed and revised the manuscript. KS and NM provided the whole-exome sequencing findings and revised the manuscript. All authors approved the final case report as submitted and agree to be accountable for all aspects of the work.

### Conflict of Interest

The authors declare that the research was conducted in the absence of any commercial or financial relationships that could be construed as a potential conflict of interest.
